# Cell Counting and Viability Assessment of 2D and 3D Cell Cultures: Expected Reliability of the Trypan Blue Assay

**DOI:** 10.1186/s12575-017-0056-3

**Published:** 2017-07-20

**Authors:** Filippo Piccinini, Anna Tesei, Chiara Arienti, Alessandro Bevilacqua

**Affiliations:** 1 Istituto Scientifico Romagnolo per lo Studio e la Cura dei Tumori (IRST) IRCCS, Via Piero Maroncelli 40, 47014 Meldola, FC Italy; 2 Advanced Research Center on Electronic Systems “Ercole De Castro” (ARCES), University of Bologna, Via Toffano 2/2, 40125 Bologna, Italy; 3 Department of Computer Science and Engineering (DISI), University of Bologna, Viale Risorgimento, 2, 40136 Bologna, Italy

**Keywords:** Microscopy, Oncology, Cell viability, Haemocytometer, Statistical analysis

## Abstract

**Background:**

Whatever the target of an experiment in cell biology, cell counting and viability assessment are always computed. The Trypan Blue (TB) assay was proposed about a century ago and is still the most widely used method to perform cell viability analysis. Furthermore, the combined use of TB with a haemocytometer is also considered the standard approach to estimate cell population density. There are numerous research articles reporting the use of TB assays to compute cell number and viability of 2D and 3D cultures. However, the literature still lacks studies regarding the reliability of the TB assay in terms of assessment of its repeatability and reproducibility.

**Methods:**

We compared the TB assay's measurements obtained by two biologists who analysed 105 different samples in double-blind for a total of 210 counts performed. We measured: (*a*) the repeatability of the count performed by the same operator; (*b*) the reproducibility of counts performed by the two operators.

**Results:**

There were no significant differences in the results obtained with 2D and 3D cell cultures: we estimated an approximate variability of 5% when the TB assay was used to assess the viability of the culture, and a variability of around 20% when it was used to determine the cell population density.

**Conclusions:**

The main aim of this study was to make researchers aware of potential measurement errors when TB is used with a haemocytometer for counting and viability measurements in 2D and 3D cultures. We believe that these results can help researchers to determine whether the expected reliability of the TB assay is compliant with their applications.

## Background

The evaluation of cell population density (i.e. the total number of living cells in the culture) and cell viability (i.e. the percentage of living cells in the sample) is fundamental during biology studies [1]. The majority of laboratories engaged in cell biology routinely perform cell viability and counting analysis for different purposes, ranging from ecosystem investigation [2] to proliferation studies [3], in both 2D (two-dimensional) [4] and 3D (three-dimensional) cell cultures [5].

Among the various typologies of 3D cell cultures, multicellular tumour spheroids are those typically used for testing drugs and radiation treatments [6]. The measurement of viability and the reduction of cancer culture population are fundamental parameters for evaluating the efficacy of the treatments under investigation [7]. Accordingly, the reliability of the method used to estimate these parameters plays a key role in this analysis [8]. In addition, cell counting and viability assessment often need to be performed for other 3D cell cultures, such as stem cell spheroids generated for regenerative medicine purposes [9], and organoids used to study (some) organ characteristics [10].

**Fig. 1:**
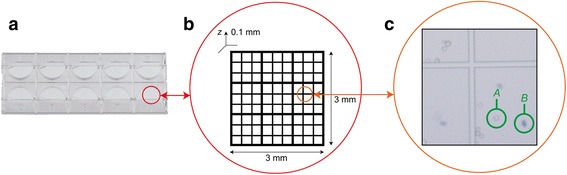
Haemocytometer grid containing cells stained with TB. **a** Picture of a Kova glasstic slide with grids (Hycor Biomedical Inc.). Each slide contains 10 counting chambers. **b** Schematic representation of the grid of a counting chamber. **c** Cells in brightfield are characterized by very low contrast. This magnified real-world detail shows some living and dead cells. In particular: **a** and **b** show the typical appearance of a living and a dead cell (stained with TB), respectively

Many different methods (e.g. AlamarBlue^®^ and MMT assay) and systems (e.g. Bio-Rad TC20™ Automated Cell Counter, ChemoMetec NucleoCounter^®^, Beckman Coulter Vi-CELL™ XR Cell Viability Analyzer [11]) can be used to analyse cell viability [12]. Most of these share the same approach: the cells are stained using a light (or a fluorescent) dye to highlight dead cells (or living cells), and a detection system counts the number of cells highlighted, in addition to the total number of cells. Finally, cell viability is computed as the percentage of healthy cells in the sample [13]. However, the Trypan Blue (TB) dye exclusion assay [14] ,the first method proposed in the literature, is considered the standard cell viability measurement method [15] and is still the most widely used approach [16]. Furthermore, TB paired with a haemocytometer grid (Fig. 1) is regarded as the standard approach for estimating the cell population density [17], i.e. the total number of living cells in the culture [18].

TB was synthesised for the first time in 1904 by Paul Ehrlich (Nobel prize in medicine, 1908) and was first used for clinical analysis before becoming a standard probe in biology. Today it is still widely used for several medical purposes such as the visualization of the lymph-associated primo vascular system [19] and of the anterior capsule during cataract surgery [20]. Chemically, TB is defined as toluidine-derived dye characterized by a molecular weight of 960 Da [15]. Its chemical construction is *C*_*34*_ *H*_*28*_ *N*_*6*_ *O*_*14*_ *S*_*4*_. Azidine Blue, Benzamine Blue, Chlorazol Blue, Diamine Blue, and Niagara Blue are synonyms for TB. TB is a cell membrane-impermeable molecule and therefore only enters cells having compromised membrane. From a practical point of view, with TB the cell viability is determined indirectly by detecting cell membrane integrity [21]. Upon entry into the cell, TB binds to intracellular proteins and in brightfield the dead cells appear blue (apoptotic and necrotic cells are not distinguished [1]), whereas the colour of living cells remains unchanged (Fig. 1c).

Over the past two decades a number of studies comparing TB with other assays have been published [15] and several methods have proven more efficient than TB [22], especially those using fluorescent dyes [23]. The use of TB has, in fact, several drawbacks [24]: (*a*) TB exerts a toxic effect on cells after a short exposure period, thus limiting cell counting to only a brief period after staining [25]; (*b*) As TB binds to cellular proteins, there is a potential for binding to non-specific cellular artifacts, especially in primary cells from clinical samples; (*c*) There is a large number of false positives, i.e. “dead cells” resulting from irreversible damage to their membrane, and false negatives from cells that have already initiated the apoptotic pathway but still have intact membranes; (*d*) There is no standardized TB concentration for the measurement of cell viability; (*e*) Manual counting using a haemocytometer and a light microscope is time-consuming and operator-dependent. Although the TB assay requires the use of a fluorescence microscope, it has long been known that several fluorescent dyes are more reliable indicators of cell viability than the more traditional coloured dyes [26]. For example, Acridine Orange (AO) and Propidium Iodide (PI) stainings have been shown to be more accurate in detecting live and dead cells than TB [27]. AO is a membrane-permeable cationic dye that binds to nucleic acids of viable cells. At low concentrations it causes a green fluorescence. PI is impermeable to intact membranes but readily penetrates the membranes of nonviable cells and binds to DNA or RNA, causing orange fluorescence. When AO and PI are used simultaneously, viable cells fluoresce green and nonviable cells fluoresce orange under fluorescence microscopy. Notwithstanding, TB is still the most commonly used dye for cell viability analysis because it is inexpensive, easy to use, it reacts quickly, and can be visualized with a standard brightfield microscope available in all biological laboratories [2]. TB is also used in several automatic counters [28] and as the reference method for comparing customized cell-counting algorithms [29]. However, in-depth validation studies of the TB assay used in combination with a haemocytometer in viability and counting measurements are lacking. Several articles have provided statistical analyses on its reliability. In 1964, Tennant [30] and Hathaway et al. [31] performed preliminary studies comparing TB, eosin Y and AO for the determination of the viability of in vitro and in vivo cultures. Twenty years later, Jones and Senft [26] also considered fluorescein diacetase (FDA) and PI. In 1999, Leite et al. [32] extended the research into this area, comparing the reliability of TB, AO and six other methods (i.e. Giemsa staining, ethidium bromide, PI, Annexin V, TUNEL assay and DNA ladder). In 2000, Mascotti et al. [27] published an in-depth comparison between AO/PI and TB assays in which the viability of 7 aliquots of hematopoietic progenitor cells (HPC) and the percentage of viable cells was calculated as the average of 5 viability measurements performed by two operators. However, as the raw counting data was not reported, it was not possible to quantitatively infer the repeatability (intra-rater reliability) and reproducibility (inter-rater reliability) of the counts. The first study on the repeatability and reproducibility of the TB assay appeared in 2011 when Sanfilippo et al. [33] assessed the reliability of TB and calcein AM/ethidium homodimer-1 (CaAM/EthD-1) staining in fresh and thawed human ovarian follicles. Measurements were performed by two independent operators. Reliability was evaluated by the intraclass correlation coefficient (ICC) and the differences between paired measurements were tested by the Wilcoxon signed-rank test. TB proved to be the more reliable staining method to evaluate follicle viability. However, the operators only evaluated 10 samples simultaneously. Finally, in 2015 Cadena-Herrera et al. [34] validated a manual, semi-automated, and fully automated TB exclusion-based methods. A single operator counted several samples in triplicate and the results obtained did not reveal a significant difference between the automated methods and the manual assay. However, 3D cell cultures were not taken into account and no considerations about measurement errors between different operators were made.

In this work we studied repeatability and reproducibility with the specific aim of assessing measurement errors occurring when TB is used in counting and viability applications in 2D and 3D cell cultures. *Repeatability* is the closeness of the agreement among subsequent measurements of the same object carried out under the same measurement conditions. *Reproducibility* is defined as the closeness of the agreement among measurements of the same object carried out under different measurement conditions [35]. In particular, the viability and total number of living cells of the culture were the “objects” being measured in our experiments. Thus, the operators performing the measurements represented the changing “condition” when assessing reproducibility. In practical terms, each operator generated and analysed 5 different samples from the same 13 2D cell cultures and 8 3D cell cultures (i.e. multicellular spheroids), making a total of 10 samples considered for each culture. Repeatability for each culture was evaluated by calculating the variability of the measurements obtained by the single operator. Conversely, reproducibility for each culture was estimated by comparing the measurements obtained by two operators. Overall, 210 samples were analysed (Table 1).

**Table 1:** Original measurements for all *S*_*k*_ analysed by *O*_*1*_ and *O*_*2*_

		*O*_*1*_			*O*_*2*_		
		Live cells	Dead cells	Viability [%]	Live cells	Dead cells	Viability [%]
*A*_*1*_	*S*_*1*_	271	39	87.42	306	33	90.27
	*S*_*2*_	330	51	86.61	339	41	89.21
	*S*_*3*_	327	37	89.84	297	28	91.38
	*S*_*4*_	363	24	93.80	345	23	93.75
	*S*_*5*_	336	40	89.36	394	30	92.92
*A*_*2*_	*S*_*1*_	234	92	71.78	325	77	80.85
	*S*_*2*_	178	57	75.74	320	71	81.84
	*S*_*3*_	176	48	78.57	274	53	83.79
	*S*_*4*_	250	67	78.86	204	55	78.76
	*S*_*5*_	442	102	81.25	244	50	82.99
*A*_*3*_	*S*_*1*_	277	114	70.84	218	79	73.40
	*S*_*2*_	259	108	70.57	241	87	73.48
	*S*_*3*_	297	111	72.79	309	101	75.37
	*S*_*4*_	253	76	76.90	220	182	54.73
	*S*_*5*_	247	86	74.17	178	64	73.55
*A*_*4*_	*S*_*1*_	248	84	74.70	364	137	72.65
	*S*_*2*_	326	121	72.93	390	136	74.14
	*S*_*3*_	173	53	76.55	407	133	75.37
	*S*_*4*_	303	105	74.26	343	119	74.24
	*S*_*5*_	301	106	73.96	364	122	74.90
*A*_*5*_	*S*_*1*_	131	119	52.40	202	145	58.21
	*S*_*2*_	130	113	53.50	218	227	48.99
	*S*_*3*_	143	64	69.08	110	24	82.09
	*S*_*4*_	166	64	72.17	172	49	77.83
	*S*_*5*_	166	83	66.67	259	68	79.20
*A*_*6*_	*S*_*1*_	91	12	88.35	162	88	64.80
	*S*_*2*_	46	35	56.79	116	76	60.42
	*S*_*3*_	81	33	71.05	83	40	67.48
	*S*_*4*_	93	49	65.49	100	48	67.57
	*S*_*5*_	101	50	66.89	128	60	68.09
*A*_*7*_	*S*_*1*_	198	206	49.01	108	103	51.18
	*S*_*2*_	244	267	47.75	165	126	56.70
	*S*_*3*_	208	163	56.06	249	190	56.72
	*S*_*4*_	207	130	61.42	177	146	54.80
	*S*_*5*_	146	120	54.89	201	174	53.60
*A*_*8*_	*S*_*1*_	111	181	38.01	142	200	41.52
	*S*_*2*_	147	294	33.33	121	220	35.48
	*S*_*3*_	178	179	49.86	199	220	47.49
	*S*_*4*_	169	137	55.23	129	142	47.60
	*S*_*5*_	147	118	55.47	106	128	45.30
*P*_*1*_	*S*_*1*_	107	11	95.24	100	5	90.68
	*S*_*2*_	80	8	96.25	77	3	90.91
	*S*_*3*_	101	9	95.18	79	4	91.82
	*S*_*4*_	83	7	95.59	65	3	92.22
	*S*_*5*_	70	6	95.65	88	4	92.11
*P*_*2*_	*S*_*1*_	106	17	86.87	86	13	86.18
	*S*_*2*_	118	21	90.00	99	11	84.89
	*S*_*3*_	99	12	87.60	106	15	89.19
	*S*_*4*_	107	12	80.00	80	20	89.92
	*S*_*5*_	119	14	78.50	84	23	89.47
*P*_*3*_	*S*_*1*_	63	14	77.61	52	15	81.82
	*S*_*2*_	46	14	74.14	43	15	76.67
	*S*_*3*_	52	10	81.69	58	13	83.87
	*S*_*4*_	75	17	72.73	56	21	81.52
	*S*_*5*_	52	11	75.86	44	14	82.53
*P*_*4*_	*S*_*1*_	55	48	54.17	39	33	53.40
	*S*_*2*_	57	44	43.48	30	39	56.44
	*S*_*3*_	49	44	51.04	49	47	52.69
	*S*_*4*_	40	30	55.65	69	55	57.14
	*S*_*5*_	38	42	57.43	85	63	47.50
*P*_*5*_	*S*_*1*_	14	116	11.59	8	61	10.77
	*S*_*2*_	13	91	9.26	5	49	12.50
	*S*_*3*_	15	127	16.22	12	62	10.56
	*S*_*4*_	18	138	10.26	8	70	11.54
	*S*_*5*_	11	71	13.33	10	65	13.41
*SP*_*1*_	*S*_*1*_	100	69	59.17	133	82	61.86
	*S*_*2*_	116	106	52.25	94	72	56.63
	*S*_*3*_	136	88	60.71	72	39	64.86
	*S*_*4*_	116	87	57.14	100	40	71.43
	*S*_*5*_	163	96	62.93	80	45	64.00
*SP*_*2*_	*S*_*1*_	155	120	56.36	66	73	47.48
	*S*_*2*_	125	94	57.08	125	71	63.78
	*S*_*3*_	158	87	64.49	103	74	58.19
	*S*_*4*_	154	75	67.25	85	68	55.56
	*S*_*5*_	156	81	65.82	219	177	55.30
*SP*_*3*_	*S*_*1*_	167	42	79.90	117	18	86.67
	*S*_*2*_	191	40	82.68	97	13	88.18
	*S*_*3*_	128	41	75.74	180	23	88.67
	*S*_*4*_	109	39	73.65	113	21	84.33
	*S*_*5*_	146	34	81.11	130	22	85.53
*SP*_*4*_	*S*_*1*_	101	71	58.72	58	33	63.74
	*S*_*2*_	114	65	63.69	163	61	72.77
	*S*_*3*_	92	60	60.53	141	45	75.81
	*S*_*4*_	92	53	63.45	124	60	67.39
	*S*_*5*_	179	77	69.92	121	56	68.36
*SP*_*5*_	*S*_*1*_	260	96	73.03	140	57	71.07
	*S*_*2*_	207	88	70.17	282	45	86.24
	*S*_*3*_	232	64	78.38	173	53	76.55
	*S*_*4*_	192	56	77.42	209	53	79.77
	*S*_*5*_	263	75	77.81	69	24	74.19
*SP*_*6*_	*S*_*1*_	222	65	77.35	175	41	81.02
	*S*_*2*_	226	66	77.40	229	59	79.51
	*S*_*3*_	216	53	80.30	108	29	78.83
	*S*_*4*_	218	54	80.15	135	37	78.49
	*S*_*5*_	205	44	82.33	254	43	85.52
*SP*_*7*_	*S*_*1*_	134	101	57.02	159	93	63.10
	*S*_*2*_	161	128	55.71	235	124	65.46
	*S*_*3*_	151	134	52.98	83	70	54.25
	*S*_*4*_	180	106	62.94	134	97	58.01
	*S*_*5*_	190	119	61.49	91	78	53.85
*SP*_*8*_	*S*_*1*_	146	197	42.57	67	105	38.95
	*S*_*2*_	178	221	44.61	110	144	43.31
	*S*_*3*_	110	159	40.89	188	241	43.82
	*S*_*4*_	68	120	36.17	124	171	42.03
	*S*_*5*_	157	214	42.32	127	154	45.20

The main aim of this work was to make researchers aware of the measurement errors that can occur when the TB assay is used to evaluate population and viability of 2D and 3D cell cultures. Given that this is a preliminary study, global accurate overall accuracy values of assay reliability used in different contexts and with different cell lines cannot be provided. However, we believe that our findings can help researchers to evaluate whether the *expected* repeatability and reproducibility of the TB assay are compliant with those required by their own application.

## Methods

### 2D Cell Cultures

To assess the TB reliability we prepared 8 25-cm^2^ flasks (called *A*_*i*_, *i* = 1, …, 8) containing A549 cells (cells at the 36th passage) and 5 25-cm^2^ flasks (called *P*_*k*_, *k* = 1, …, 5) containing PANC-1 cells (cells at the 116th passage). A549 and PANC-1 are well known and widely used commercial cancer cell lines (American Type Culture Collection - ATCC, Rockville, MD, USA). A549, a lung adenocarcinoma cell line of regular-shaped cells, was adhesion-cultured in Kaighn’s modification of Ham’s F-12 medium (F12 K, ATCC) and supplemented with 10% fetal bovine serum (FBS, EuroClone, Milan, Italy), 1% penicillin/streptomycin (GE Healthcare, Milan, Italy) and 2% amphotericin B (Euroclone). PANC-1, an epithelioid cell line derived from a human pancreatic carcinoma of ductal cell origin, was grown in medium composed of DMEM/Ham’s F12 (1:1) (Euroclone) supplemented with 10% fetal calf serum (FCS, Euroclone), 2 mM glutamine (Euroclone) and 10 mg/ml insulin (Sigma-Aldrich, St. Louis, MO, USA). All the cells were maintained in an incubator at 5% CO_2_ humidified atmosphere at 37 °C and checked periodically for mycoplasma contamination using the MycoAlertTM Mycoplasma Detection Kit (Lonza, Basel, Switzerland). Once detached from the surface of the flask, cells started losing their morphology and gradually became round.

All flasks *Ai* were prepared simultaneously in the morning and kept in the incubator for 24 h. Then, as previously done by Cadena-Herrera et al. [34], each flask *A*_*i*_ was subjected to a different thermal shock to differentiate the cell viability between flasks. *A*_*1*_ and *A*_*2*_ were simply moved from the incubator to a sterile laminar flow hood at room temperature*. A*_*3*_ and *A*_*4*_ underwent a freeze-thaw cycle (incubator at 37 °C, freezer at −80 °C and were then returned once to the incubator at 37 °C). *A*_*5*_ and *A*_*6*_ underwent the same procedure twice, and *A*_*7*_ and *A*_*8*_ *,* three times. For each freeze-thaw cycle, *A*_*3*_, *A*_*5*_ and *A*_*7*_ were kept in the freezer for 15 min, and *A*_*4*_, *A*_*6*_ and *A*_*8*_ for 30 min. Of note, the thermal shocks were carried out sequentially in the morning and the counting measurements were performed for all the flasks in the afternoon of the same day.

We used gemcitabine, a well known chemotherapeutic agent used to treat several tumours, including pancreatic cancer [36], to modulate the viability of the cells contained in the different *P*_*k*_. All *P*_*k*_ were prepared simultaneously on the same morning and gemcitabine was tested at scalar concentrations of 5 μM (flask *P2*), 50 μM (*P3*), 500 μM (*P4*), and 1000 μM (*P5*). *P1* contained untreated cells. An exposure time of 1 h followed by a 72-h wash out was chosen on the basis of peak plasma levels defined in recent pharmacokinetic studies [37].

### 3D Cell Cultures

**Fig. 2:**
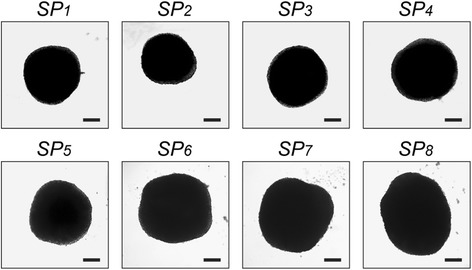
Multicellular cancer spheroids obtained from lung cancer cells (line A549), built using a RCCS-8DQ bioreactor (Synthecon Inc., Houston, TX, USA). Scale bar 200 μm

The A549 cells described in Section 2.1 were also used to produce the multicellular spheroids. Several systems and methods are available to generate in vitro multicellular spheroids of different dimensions [38]. We used a rotatory cell culture system, the RCCS-8DQ bioreactor (Synthecon Inc., Houston, TX, USA), which is capable of controlling up to 4 rotating chambers, even at different speeds. The rotator bases were placed inside a humidified, 37 °C, 5% CO_2_ incubator and connected to power supplies on the external side of the incubator. All activities were performed in sterile conditions under a laminar flow hood, as previously described [7]. Briefly: a single cell suspension of about 1 × 10^6^ cells/ml was placed in a single 50-ml rotating chamber at an initial speed of 12 rpm (rpm), increasing as the size of the spheroids increased to avoid aggregate sedimentation within the culture vessels. The culture medium was changed every 4 days. After 15 days the spheroids had reached a diameter of 0.5–1 mm and were transferred (one spheroid/well) under a sterile laminar flow hood to 96-well low-attachment culture plates (Corning Inc., Corning, NY, USA), each well previously filled with 100 μl of fresh culture medium. After the *spheroidization time* (i.e. 1 week [7]), each spheroid was imaged in brightfield using an inverted Olympus IX51 widefield microscope equipped with an Olympus UPlanFl 4×/0.13na as a standard objective lens and endowed with a Nikon Digital SightDS-Vi1 camera (CCD vision sensor, square pixels of 4.4 μm side length, 1600 × 1200 pixel resolution, 3-channel images, 8-bit grey level). For spheroids with partially out-of-focus borders, we acquired a *z*-stack of brightfield images and reconstructed a single 2D image fully in-focus by using the open-source tool previously described [39]. We then vignetting corrected the images with *CIDRE* [40], segmented the spheroids using *AnaSP* [41], and computed their volume by *ReViSP* [42, 43]. To assess TB reliability, eight compact spheroids with regular shape but a different volume (called *SP*_*i*_, *i* = 1, …, 8, Fig. 2) were transferred to a different plate and digested into single cells using a Trypsin/EDTA 1× solution (Euroclone, Milan, Italy) [44].

### Sample Preparation

We used a haemocytometer (Kova glasstic slide with grids, Hycor Biomedical Inc., Fig. 1b) and a commercially available TB preparation (TB solution 0.4%, SIGMA-ALDRICH, Buchs, Switzerland) to perform the counts. A detailed description of the protocol adopted with TB is reported in [11, 21] and [45]. In brief, for each *Ai* we:

1. detached the cells from the flask by trypsinization;
2. centrifuged the cell suspension for 5 min at 1200 rpm;
3. resuspended the pellet in 1 ml of culture media using a pipette to obtain a single-cell suspension;
4. removed an aliquot of 100 μl;
5. added 100 μl of TB solution 0.4% to obtain a final 1:2 dilution;
6. waited for 5 min to allow the TB to stain the dead cells;
7. counted the cells using a haemocytometer and a light microscope;
8. calculated the percentage of viability and number of cells in the culture by considering the final dilution factor.

We followed the same protocol for the different *P*_*k*_ but used a 1:6 dilution. For the different *SPi* we used the same protocol as that used for *Ai* but with the pellet resuspended in 200 μl of culture media (not 1 ml, as described in point *3*).

Two expert operators (hereafter *O*_*1*_ and *O*_*2*_) performed a double-blind evaluation of the viability and population of a set of 5 single-cell suspensions (*S*_*k*_, *k* = 1, …, 5) for each *A*_*i*_, *P*_*k*_ and *SP*_*i*_; making a total of 210 samples analysed. Of note, both *O*_*1*_ and *O*_*2*_ prepared their own suspensions for each *A*_*i*_/*P*_*k*_ */SP*_*i*_. Using a Falcon 2 ml serological pipet for each *S*_*k*_ they gently pipetted up and down 30 times in about 15 s to disaggregate all the possible cell clumps before loading a drop into a counting chamber. Differences in viability due to different cultivation/waiting times were avoided by simultaneously counting the samples of the same flask/spheroid in double blind. In particular, the operators used two widefield microscopes with similar optics, located in the same room and used daily for counting applications. The first was an inverted Olympus IX51 widefield microscope equipped with an Olympus UPlanFl 10×/0.30na Ph1 objective infinity corrected, while the second was an inverted Zeiss Axiovert 200 widefield microscope equipped with a Zeiss Achroplan 10×/0.25na Ph1 objective infinity corrected. Both microscopes were used in brightfield, and the Köhler illumination alignment [46] was performed in advance.

### Sources of Error for Counting Measurements

Several sources of error contributed to the variability in the counts performed with the TB assay and can be summarized as follows (https://chemometec.com/manual-cell-counting/):

1. Subjective definition of a “cell”: There are guidelines but no well defined rules to help an operator define a cell. From a practical point of view, distinguishing a cell from cell debris or other particles is often challenging, even for an expert biologist.
2. Subjective perception of a “dead cell”: With TB there is no official colour threshold for discriminating between a dead cell and a living one. Individual operators performing the manual count has a certain specific set of criteria to define the threshold of brightness of the stain in order to count a cell as being viable or not. Such interpersonal differences in the manual identification of dead cells are crucial for defining the percentage of viability of the cell culture.
3. Dilution and pipetting errors: The final sample of cells to be counted is the result of several dilutions of the original cell culture. Small pipetting errors substantially influence the final estimation of the cell population density because they concatenate and contribute to the end result as multiplicative factors.
4. Time per sample: Counting cells at the microscope is tedious and time-consuming. In addition, and cells die due to the cytotoxic effect of TB and so, all the samples should be analysed at exactly the same time. However, standardization of the counting time is not possible because it is based on the number of cells in the sample.
5. Samples with a “right” number of cells: Even a few mismatches of dead cells can strongly influence the final evaluation of culture viability if the sample analysed with the haemocytometer contains a low number of cells. On the other hand, samples containing too high a number of cells can can lead to an incorrect estimation of cell population density because it is difficult to remember the cells that have been counted when using a haemocytometer with a grid that has only a few reference lines.

### Statistical Analysis

The reproducibility and repeatability of the TB assay was measured by analysing the 210 counts performed by *O*_*1*_ and *O*_*2*_. In particular, for cell viability we computed the mean and standard deviation (i.e., μ and σ values of the different *S*_*k*_) of the percentage of living cells estimated by *O*_*1*_ and *O*_*2*_ for each *A*_*i*_ (results reported in Table 2), *P*_*k*_ (Table 5) and *SP*_*i*_ (Table 8). As for the cell population density assessment, we estimated the mean and coefficient of variation (i.e., μ and CV of the different *S*_*k*_) of the total number of living cells for each *A*_*i*_ (Table 3), *P*_*k*_ (Table 6) and *SP*_*i*_ (Table 9). Specifically, we first computed μ and σ of the 5 *S*_*k*_ analysed by each operator for each *A*_*i*_/*P*_*k*_/*SP*_*i*_, and then computed the CV values. Finally, we calculated the absolute percentage error (*E*%) of the values obtained by the two operators, defined according to Eq. 1:

1 $$ E\%=\left|\frac{v_1-{v}_2}{v_{12}}\right|\cdot 100. $$

**Table 2:** Cell viability (μ and σ) estimated by *O*_*1*_ and *O*_*2*_ for the different *A*_*i*_

	Percentage of living cells [%]				*p*-value
	O_1_		O_2_		
	μ	σ	μ	σ	
A_1_	89.41	2.79	91.51	1.86	0.31
A_2_	77.24	3.62	81.65	1.96	0.06
A_3_	73.06	2.61	70.10	8.64	1.00
A_4_	74.48	1.33	74.26	1.03	1.00
A_5_	62.76	9.18	69.26	14.75	0.42
A_6_	69.71	11.64	65.67	3.20	0.84
A_7_	53.83	5.57	54.60	2.32	0.84
A_8_	46.38	10.16	43.48	5.10	0.55
Average	//	5.86	//	4.86	

*μ* mean*,* σ standard deviation

**Table 3:** Cell population density (μ and CV) estimated by *O*_*1*_ and *O*_*2*_ for the different *A*_*i*_

	Total number of living cells				*p*-value
	O_1_		O_2_		
	μ	CV [%]	μ	CV [%]	
A_1_	325	10.32	336	11.40	0.69
A_2_	256	42.61	273	18.75	0.42
A_3_	267	7.64	233	20.63	0.15
A_4_	270	22.72	373	6.70	0.01
A_5_	147	12.17	192	28.96	0.13
A_6_	82	26.16	118	25.43	0.10
A_7_	201	17.57	180	28.63	0.55
A_8_	150	17.22	139	25.67	0.38
Average	//	19.55	//	20.77	

μ mean*, CV* coefficient of variation

For cell viability and total number of living cells, *v*_1_ and *v*_2_ are the mean values estimated by *O*_*1*_ and *O*_*2*_, respectively, while *v*_12_ is the mean value estimated considering all 10 samples for each *A*_*i,*_/*P*_*k*_/*SP*_*i*_ analysed by the two operators. Finally, a two-sided Wilcoxon rank-sum test was used to compare the values obtained by the different operators for both cell viability and total number of living cells. MATLAB (©, The MathWorks, Inc., Natick, Massachusetts, USA) was used for statistical analysis. *p*-values < 0.05 were considered significant. The results obtained from the *Ai* analysis are reported in Tables 2, 3, and 4. Tables 5, 6, and 7 report the results for *P*_*k*_, and Tables 8, 9, and 10 show the results for *SPi*.

**Table 4:** *E*% computed between the μ value estimated by *O*_*1*_ and *O*_*2*_ for the different *A*_*i*_

	E%	
	Percentage of living cells [%]	Total number of living cells
A_1_	2.32	3.26
A_2_	5.55	6.57
A_3_	4.13	13.37
A_4_	0.29	32.12
A_5_	9.85	26.52
A_6_	5.98	35.36
A_7_	1.43	10.83
A_8_	6.46	7.59
Average	4.50	16.95

*E%* absolute percentage error

**Table 5:** Cell viability (μ and σ) estimated by *O*_*1*_ and *O*_*2*_ for the different *P*_*k*_

	Percentage of living cells [%]				*p*-value
	O_1_		O_2_		
	μ	σ	μ	σ	
P_1_	91.55	0.71	95.58	0.43	0.01
P_2_	87.93	2.23	84.60	5.04	0.55
P_3_	81.28	2.74	76.41	3.48	0.06
P_4_	53.43	3.83	52.35	5.49	1.00
P_5_	11.75	1.20	12.13	2.74	1.00
Average	//	2.14	//	3.44	

μ mean*,* σ standard deviation

**Table 6:** Cell population density (μ and CV) estimated by *O*_*1*_ and *O*_*2*_ for the different *P*_*k*_

	Total number of living cells				*p*-value
	O_1_		O_2_		
	μ	CV [%]	μ	CV [%]	
P_1_	88.20	17.41	81.80	15.97	0.42
P_2_	109.80	7.77	91.00	12.09	0.04
P_3_	57.60	19.97	50.60	13.52	0.42
P_4_	47.08	17.96	55.40	41.22	0.88
P_5_	14.20	18.23	8.60	30.32	0.02
Average	//	16.27	//	22.62	

μ mean*, CV coefficient of variation*

**Table 7:** *E*% computed between the μ value estimated by *O*_*1*_ and *O*_*2*_ for the different *P*_*k*_

	E%	
	Percentage of living cells [%]	Total number of living cells
P_1_	4.31	7.53
P_2_	3.86	18.73
P_3_	6.18	12.94
P_4_	2.04	16.28
P_5_	3.18	49.12
Average	3.91	20.91

*E%* absolute percentage error

**Table 8:** Cell viability (μ and σ) estimated by *O*_*1*_ and *O*_*2*_ for the different *SP*_*i*_

	Percentage of living cells [%]				*p*-value
	O_1_		O_2_		
	μ	σ	μ	σ	
SP_1_	58.44	4.06	63.76	5.35	0.15
SP _2_	62.20	5.10	56.06	5.88	0.10
SP _3_	78.62	3.79	86.67	1.81	0.01
SP _4_	63.26	4.26	69.61	4.73	0.06
SP _5_	75.36	3.60	77.56	5.80	0.69
SP _6_	79.50	2.13	80.68	2.88	0.69
SP _7_	58.02	4.12	58.93	5.21	0.69
SP _8_	41.31	13.05	42.66	2.36	0.54
Average	//	5.01	//	4.25	

μ mean*,* σ standard deviation

**Table 9:** Cell population density (μ and CV) estimated by *O*_*1*_ and *O*_*2*_ for the different *SP*_*i*_

	Total number of living cells				*p*-value
	O_1_		O_2_		
	μ	CV [%]	μ	CV [%]	
SP _1_	126	19.19	96	24.60	0.07
SP _2_	150	9.25	120	49.91	0.17
SP _3_	148	21.69	127	24.86	0.42
SP _4_	116	31.63	121	32.28	0.50
SP _5_	231	13.64	175	45.34	0.31
SP _6_	217	3.65	180	34.10	0.69
SP _7_	163	13.72	140	43.72	0.33
SP _8_	132	32.89	123	35.26	0.74
Average	//	18.21	//	36.26	

μ mean*, CV* coefficient of variation

**Table 10:** *E*% computed between the μ value estimated by *O*_*1*_ and *O*_*2*_ for the different *SP*_*i*_

	E%	
	Percentage of living cells [%]	Total number of living cells
SP _1_	8.70	27.38
SP _2_	10.38	22.29
SP _3_	9.75	15.09
SP _4_	9.56	4.89
SP _5_	2.88	27.73
SP _6_	1.46	18.71
SP _7_	1.55	15.01
SP _8_	3.22	6.74
Average	5.94	17.23

*E%* absolute percentage error

## Results

### Analysis of the 2D Cell Cultures

We used the σ values obtained for *A*_*i*_ and *P*_*k*_ to estimate the intra-rater reliability of cell viability (Tables 2 and 5, respectively). Given that cell viability is computed as a percentage, the standard deviation can be considered a direct estimation of the error that may occur when TB is used to estimate cell viability. All σ values were lower than 15% for both *O*_*1*_ and *O*_*2*_. Furthermore, the average σ values were approximately 5% for *A*_*i*_ and 3% for *P*_*k*_ (*last row* of Table 2 and Table 5, respectively), indicating the high reliability of the TB assay when used for this purpose. With regard to the inter-rater reliability of cell viability we considered the *E*% values reported in the *second column* of Tables 4 and Table 7. It is worthy of note that the mean cell viability values estimated by *O*_*1*_ and *O*_*2*_ for each *A*_*i*_/*P*_*k*_ were fairly similar (from left, the *second* and the *forth column* of Table 2 and Table 5). Accordingly, *E*% values reported in Table 4 and Table 7 were very low, i.e. <10%, and their average was <5% (*last row*, *second column* of Table 4 and Table 7).

Conversely, both the intra- and inter-rater variability values obtained for the total amount of living cells were particularly high. Being the total amount of cells computed as the absolute value, we estimated the intra-rater variability by analysing the CV values for all *A*_*i*_/*P*_*k*_, considering the different *S*_*k*_ counted by the operators. The majority of CVs reported in Table 3 and Table 6 were >15%, which is fairly surprising. In particular, *O*_*1*_ obtained a CV <10% twice (i.e. for *A*_*3*_ and *P*_*2*_) and *O*_*2*_ only once (i.e. for *A*_*4*_). Furthermore, the average CV values (*bottom row* of Table 3 and Table 6) were particularly high (around 20%) for both operators. Similarly, as the amount of living cells estimated by *O*_*1*_ and *O*_*2*_ for each *A*_*i*_/*P*_*k*_ differed substantially (*second* and *forth column* of Table 3 and Table 6), the majority of *E*% values reported in the *third column* of Table 4 and Table 7 were especially high. In particular, the average *E%* (*bottom row, right-hand column* of Table 4 and Table 7) was >15% for both *A*_*i*_ and *P*_*k*_. These results, paired with the previously described high intra-rater variability, unexpectedly revealed a poor ability of the TB assay to estimate cell population density.

However, many of the *p*-values computed for both viability and total number of living cells were >0.05, this proving that the sets of counts obtained by *O*_*1*_ and *O*_*2*_ for the same *A*_*i*_/*P*_*k*_ did not differ significantly from each other. In actual fact they differed in one only case for *A*_*i*_ (Table 3 **,** row *A*_*4*_), and in three cases for *P*_*k*_ (Table 5 **,** row *P*_*1*_ and Table 6 **,** rows *P*_*2*_ and *P*_*5*_). The differences obtained by the two operators in these cases were probably caused by a pipetting/resuspending error. For example, the data in Table 1 clearly show that the number of cells counted by *O*_*1*_ for *A*_*4*_ was significantly lower and more variable than those counted by *O*_*2*_. However, a *p*-value <0.05 in 4 out of 26 cases simply means that, despite the high intra-rater reliability of the TB assay, especially when used for cell population density assessment, the sets of counts performed by different operators did not, in general, differ statistically.

### Analysis of the 3D Cell Cultures

The results obtained from the analysis of the 3D cell cultures were similar to those obtained for the 2D cultures. Only one *p*-value (Table 8 row *SP*_*3*_) was <0.05, which again indicates that the measurements obtained by *O*_*1*_ and *O*_*2*_ did not differ significantly.

All σ values reported in Table 8 were <15%, and the average σ were 4.84% and 4.23% for *O*_*1*_ and *O*_*2,*_ *respectively*, once more confirming the high repeatability of the TB assay when used to estimate the viability of 2D and 3D cell cultures. The *E*% values reported in the *second column* of Table 10 were slightly higher than those of Table 4 and Table 7, suggesting poorer reproducibility of cell viability values for 3D cultures (but still around 5%).

With regard to the analysis of cell population density, both intra- and inter-rater variability were once again exceptionally high. The majority of CVs reported in Table 9 were >20%, *O*_*2*_ never obtaining a CV <20%, and *O*_*1*_ only twice obtaining a value <10% (i.e. for *SP*_*2*_ and *SP*_*6*_). Similarly to what happened for the 2D A549 cell cultures, the amount of living cells estimated by *O*_*1*_ for *SP*_*i*_ differed substantially from that obtained by *O*_*2*_ (*second column* vs *forth column,* Table 9). Consequently, most of the *E*% values reported in the *third column* of Table 10 were >15%, with an average *E%* of 17.23%. Notably, the CV value obtained by *O*_*2*_ for *SP*_*2*_, *SP*_*5*_, *SP*_*6*_, *SP*_*7*_ was triple that obtained by *O*_*1*_ because the total number of living cells counted by *O*_*2*_ for these *SP*_*i*_ was much more variable than that of the counts performed by *O*_*1*_. Specifically, the σ of the counts performed by *O*_*2*_ was more than twice that of the counts performed by *O*_*1*_. Furthermore, *O*_*2*_ counted a lower number of cells than *O*_*1*_ for all but *SP*_*4*_, probably because there were more cell clusters in the samples prepared by *O*_*2*_ that must not be considered when counting with a haemocytometer (here, we remark that each operator prepared her/his own 5 *S*_*k*_). This resulted in a lower μ of the number of living cells counted by *O*_*2*_ which negatively contributed to the estimation of the CV values. Although both operators are biologists with more than 10 years’ experience in counting cells, the results are suggestive of a greater ability of *O*_*1*_ to resuspend the samples generated from 3D spheroids, effectively disgregating the cell clusters. This is indicative of the high subjectivity of the TB assay and of it poor reliability when used to estimate the total number of cells in a culture. However, as happened for the 2D cell cultures, almost all *p*-values computed for viability and total number of living cells were >0.05, once more proving that the sets of counts obtained by the different operators did not significantly differ from each other.

## Discussion

In this work we studied repeatability and reproducibility of cell population and viability measurements obtained with the TB assay. We asked two experienced biologists to count the live and dead cells of 105 different samples of 2D and 3D cell cultures in a double blind manner (total 210 counts). Our aim being to measure: (*a*) the repeatability of the count performed by the same operator; (*b*) the reproducibility of counts performed by the two operators.

We estimated an approximate variability of 5% for both 2D and 3D cell cultures when the TB assay is used to assess the viability of the culture, and a variability of around 20% when it was used to determine the cell population density, i.e. total number of living cells in the culture. Our results show that, whilst the method is quite precise when used to assess viability, it is fairly unreliable at estimating the population of a cell culture, whether 2D or 3D. In practice, our findings serve to alert researchers evaluating cell culture populations that they should expect to find an appreciable difference between measurements (up to 20%) when performed by different operators.

## Conclusions

The TB assay was introduced about a century ago and is still the most widely used method to perform viability and population assessments of cell cultures. However, no study has been published so far with regard to deep validation of the TB assay, especially for viability and counting measurements of 3D cell cultures.

The main aim of the statistical analyses performed in this work was to provide researchers with novel information on TB reliability and to make them aware of *expected* measurement errors when the assay is used to evaluate population and viability of 2D and 3D cell cultures. The results obtained prove that (*a*) there is no significant difference between 2D and 3D cell cultures as far as TB reliability is concerned; (*b*) the TB method is precise when used for viability assessments of a cell culture; (c) the method is fairly inaccurate at estimating cell population density, despite it is routinely used for this purpose in numerous laboratories.

For the sake of clarity we repeat that as mentioned before, the purpose of our work was not to provide overall accuracy of the reliability of an assay used in different contexts and with different cell lines. Nevertheless, once these performances are known and acknowledged, it will be up to researchers to determine when the TB assay can be used and whether the *expected* reliability of its measurements is compliant with their own experiments.

## Abbreviations

TB: Trypan blue
2D: Two-dimensional
3D: Three-dimensional
Da: Dalton
AO: Acridine orange
PI: Propidium iodide
FDA: Fluorescein diacetase
HPC: Hematopoietic progenitor cells
CaAM: Calcein AM
EthD-1: Ethidium homodimer-1
ICC: Intraclass correlation coefficient
ATCC: American type culture collection
F12 K: Ham’s F-12 medium
FBS: Fetal bovine serum
°C: Degree celsius
RCCS: Rotatory cell culture system
rpm: Revolutions per minute
mm: Millimetre
μl: Microlitre
CCD: Charge-coupled device
*SP*_*i*_: Spheroid *i*
*O*: Operator
na: Numerical aperture
μ: Mean
σ: Standard deviation
CV: Coefficient of variation
*E%*: Absolute percentage error
*v*_i_: Mean value estimated by *O*_*i*_
ANOVA: One-way analysis of variance
